# PHYSICAL ACTIVITY LEVEL IN ASTHMATIC ADOLESCENTS: CROSS-SECTIONAL POPULATION-BASED STUDY

**DOI:** 10.1590/1984-0462/;2019;37;2;00002

**Published:** 2019-01-07

**Authors:** Marco Aurélio de Valois Correia, Emília Chagas Costa, Laienne Carla Barbosa de Barros, Andressa Araújo Soares, Emanuel Sávio Cavalcanti Sarinho, José Angelo Rizzo, Silvia Wanick Sarinho

**Affiliations:** a Universidade de Pernambuco, Recife, PE, Brasil.; b Universidade Federal de Pernambuco, Recife, PE, Brasil.; c Universidade de Pernambuco, Petrolina, PE, Brasil.

**Keywords:** Asthma, Excercise, Adolescents, Epidemiology, Asma, Atividade física, Adolescentes, Epidemiologia

## Abstract

**Objective:**

To assess the level of physical activity in asthmatics in comparison with non-asthmatics in a population study.

**Methods:**

Cross-sectional study with 13 to 14-year-old adolescents who participated in the *International Study of Asthma and Allergies in Childhood* (ISAAC). The subjects were classified into three groups: individuals with active asthma, individuals diagnosed with asthma, and respiratory asymptomatic individuals. To evaluate the level of physical activity, the International Physical Activity Questionnaire (IPAQ-short version) was used. The analysis consisted of comparing individuals with active asthma, diagnosed as asthmatic and asymptomatic, with a significance level of 5%.

**Results:**

The participants were 1,591 adolescents, of which 791 (49.7%) were male. There were 222 (14.0%) individuals with active asthma and 284 (17.8%) asthma diagnoses; 55% of the population were physically active. Adolescents diagnosed with asthma were more active than their non-asthmatic peers (64.4 *versus* 53.3%; p=0.001).

**Conclusions:**

Adolescents diagnosed with asthma were more physically active than their non-asthmatic peers.

## RESUMO

**Objetivo:**

Avaliar o nível de atividade física (AF) em asmáticos, comparando com não asmáticos, em estudo populacional.

**Métodos:**

Estudo transversal com adolescentes de 13 a 14 anos que participaram do estudo *International Study of Asthma and Allergies in Childhood* (ISAAC). Os indivíduos foram classificados em três grupos: indivíduos com asma ativa, indivíduos com diagnóstico de asma e assintomáticos respiratórios. Para avaliar o nível de AF, foi utilizado o Questionário Internacional de Atividade Física (IPAQ) versão curta. A análise consistiu na comparação de indivíduos com asma ativa, com diagnóstico de asma e assintomáticos, sendo o nível de significância considerado de 5%.

**Resultados:**

Participaram da pesquisa 1.591 adolescentes, 791 (49,7%) do sexo masculino. Apresentaram asma ativa 222 (14,0%) indivíduos e diagnóstico de asma 284 (17,8%). Foram fisicamente ativos 55,1% da população. Adolescentes com diagnóstico de asma foram mais ativos do que seus pares não asmáticos (64,4 *versus* 53,3%; p=0,001).

**Conclusões:**

Adolescentes com diagnóstico de asma apresentavam-se fisicamente mais ativos do que seus pares não asmáticos.

## INTRODUCTION

The perception of asthma symptoms after exercise and the fear that it may trigger the disease may have some negative repercussions on the practice of physical activity (PA) in children and adolescents.^1^ ^,^ ^2^ ^,^ ^3^ However, those with asthma should be encouraged to practice physical activity in the same way as those without the disease, as the benefits are the same for both, i.e. it is through it that children are included in society and relate, whether by playing or engaging in sports activities, preventing psychological/social isolation and improving self-esteem, quality of life and aerobic conditioning. ^1^ ^,^ ^2^ ^,^ ^3^ ^,^ ^4^

In assessing the benefits of PA in asthmatics, França-Pinto et al.^5^ concluded that there was a significant improvement in severity, frequency of attacks, diurnal and nocturnal symptoms, and use of medications. In addition, they have shown that clinical treatment associated with physical activity improves self-management and symptomatology skills. However, some studies continue to show that asthmatic children practice less PA than their peers,^6^ ^,^ ^7^ ^,^ ^8^ although it is not a consensus.^3^ ^,^ ^4^ ^,^ ^9^ These data point to a possible behavioral differentiation between asthmatics and the general population regarding physical activity.

Systematic reviews and consensuses have described that low physical fitness may play a role in increasing the severity of asthma and that physical training may be very important in the treatment of these children.^4^ ^,^ ^10^ ^,^ ^11^ ^,^ ^12^ Thus, it is important to encourage adolescents to practice PA and assess whether asthmatic subjects exhibit any restrictive behavior regarding sports practices.

Several studies have shown that asthmatic children practice less PA than their peers.^4^ ^,^ ^6^ ^,^ ^7^ ^,^ ^8^ ^,^ ^13^ In this sense, this research aimed to evaluate the level of physical activity in asthmatic adolescents, in comparison with non-asthmatic patients, in a population study.

## METHOD

This is a cross-sectional study with 13 to 14-year-old adolescents from Petrolina, PE, Brazil, who participated in the International Study of Asthma and Allergies in Childhood (ISAAC).^14^ This study was approved by the Committee of Ethics in Research with human beings of Universidade de Pernambuco (Protocol no. 459.304).

All participants in the ISAAC study^14^ were included in the study and had their data evaluated according to the level of PA, allergic diseases, age, sex, maternal education, and family history of asthma. Based on the relationship with allergic diseases, subjects were classified into three groups: individuals with active asthma, individuals diagnosed asthma and respiratory asymptomatic individuals. The ISAAC questionnaire and the International Physical Activity Questionnaire (IPAQ) were distributed in classrooms and filled out by the adolescents themselves under the supervision of the previously trained researchers.

Sample selection followed the ISAAC recommendation, which suggests a minimum sample of 1,000 participants to obtain good representativeness. The procedure followed a sequence of steps in an attempt to obtain a representative sample of students from the state school system regarding the distribution according to the size of schools (population estimated at 31,555 and sample size of 1,425 students).^14^ In order to assist sample planning, schools were organized into three categories - small (up to 200 students), medium-sized (201 to 499 students) and large (over 500 students) - and the stratification criterion was applied considering proportionality by size. After all the stages, there was a total of 18 schools and 57 classes, representing 42% of the state schools in the city of Petrolina. Considering a minimum number of 25 students per class, a total of 1,425 students were expected for evaluation. As some classrooms had more than the estimated number of students, a total of 1,591 students were evaluated. The schools were chosen by lottery after randomization carried out in the WinPepi software (PEPI-for-Windows, version 7.7, New York, USA).

For the evaluation of allergic diseases, the ISAAC questionnaire, translated and validated for Brazil, was used.^15^ The questionnaire has three modules (asthma, rhinitis, and eczema) with up to eight questions each, is self-administered and easily understood. Individuals who answered “yes” to the question “Have you experienced chest wheezing in the last 12 months?” were considered to have current or active asthma. This is the question with higher sensitivity for asthma prevalence, and limits the time period to 12 months to reduce memory biases. The diagnosis of asthma was given through the question “Have you ever had asthma in your life?”. This question assesses asthma diagnosed by a physician and for the diagnosis of a history of severe attacks of the disease; adolescents who reported chest wheezing so strong as to affect speech were considered. This questionnaire does not contain questions regarding the classification of asthma between attacks or medicine intake. Those who had nor active asthma neither asthma diagnosis were classified as respiratory asymptomatic individuals.

To evaluate the level of PA, the short version of IPAQ was translated and validated for Brazil.^16^ ^,^ ^17^ The questions are related to the time the respondent spent doing PA in the last week. According to the IPAQ, people are classified into five categories: very active (those who practice vigorous PA ≥5 days/week for ≥30 minutes/session, or vigorous ≥3 days/week for ≥20 ­minutes/session+moderate and/or walk ≥5 days/week for ≥30 minutes/session); active (who practices vigorous PA ≥3 days/week for ≥20 minutes/session or moderate or walking ≥5 days/week for ≥30 minutes/session or any added activity ≥5 days/week and 150 minutes/week - walk+moderate+vigorous); irregularly active, which was divided into irregularly active A (those who practice PA 5 days/week or lasting 150 minutes/week) and irregularly active B (those who did not meet any of the recommendation criteria as to the frequency as well as duration); or sedentary (those who do not perform any PA for at least 10 continuous minutes during the week).

For analysis in this study, the individuals classified by IPAQ as very active and active were considered as “active”, those who were irregularly active and sedentary were considered as “inactive”.

Statistical analysis was performed using GraphPad Prism 4^®^ software (San Diego, USA). The values were expressed as absolute values and percentages, and the evaluation of the differences between the proportions was performed through the chi-square test, with Yates correction when necessary. All conclusions were taken at a significance level of 5%.

## RESULTS

A total of 1,591 adolescents participated in the study. Of these, 1,307 did not report any history of asthma, while 284 (17.9%) had presented asthma at any moment in life, and of these, 222 (14.0%) indicated the active version of the disease, of which 165 (10.4%) reported a history of severe attacks.

Of the total sample, 791 were males (49.7%). Only 17.7% of the students’ mothers had completed higher education, and only 6.5% earned more than five minimum salaries. Rhinitis was reported by 408 (25.6%), and eczema by 175 (11.0%) adolescents; 55.1% of the study population were physically active (Table 1).

**Table 1:** General characteristics of 1,591 adolescents who responded adequately to the standardized questionnaire of the International Study of Asthma and Allergies in Childhood.

	n	%
Males	791	49.7
Maternal schooling		
Primary education	507	31.9
Secondary education	573	36.0
Higher education	281	17.7
Not informed	230	14.5
Family income (number of minimum wages)		
Up to 1	468	29.4
1 to 2	600	37.8
3 to 5	223	14.0
More than 5	103	06.5
Not informed	197	12.4
Chest wheezing in the last 12 months	222	14.0
Wheezing fits in the last 12 months		
1-3 fits	188	84.7
4-12 fits	28	12.6
>12 fits	06	2.7
Night awakening in the last 12 months		
Never	123	55.4
<1 night/week	58	26.1
1 or more nights/week	41	18.5
“Have you ever had asthma?”	284	17.9
Difficulty speaking in the last 12 months	165	10.4
Family member with asthma	427	26.8
“Have you ever had rhinitis?”	408	25.6
“Have you ever had eczema?”	175	11.0
Physical Activity Level: Active	876	55.1

**Figure 1:**
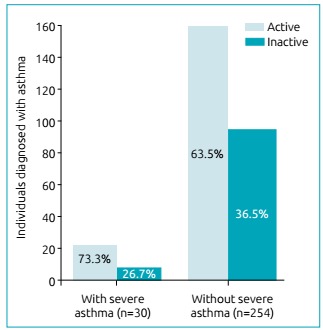
Association between physical activity level and history of severe asthma exacerbation in 284 individuals who reported having diagnosed asthma using the International Study of Asthma and Allergies in Childhood questionnaire (p=0.26)

Adolescents diagnosed with asthma were more active than their non-asthmatic peers (Table 2). Figure 1 shows that individuals with a history of severe asthma exacerbation were as active as their peers who did not have this history of severe attacks (73.3 *versus* 63.5%; p=0.26).

**Table 2:** Association between the diagnosis of asthma (ever had the disease in lifetime according to the International Study of Asthma and Allergies in Childhood), absence of wheezing in the last 12 months, prevalence of asthma and the level of physical activity in participants of the population-based study.

	Total	Physically active		
		n	%	p-value*
Diagnosis of asthma				
Yes	284	183	64.4	0.001
No	1,307	697	53.3	
Diagnosis of asthma without wheezing in the last 12 months				
Yes	62	39	62.9	0.139
No	1,307	697	53.3	
Active asthma (wheezing in the last 12 months)				
Yes	222	59	26.5	0.001
No	1,307	697	53.3	

*Chi-square test.

## DISCUSSION

This study presents divergent results from the usual literature, as it found that adolescents aged 12 to 13 years who were diagnosed with asthma at any time in their lives had higher PA levels than individuals who did not report symptoms of the disease. Individuals diagnosed with it may have been encouraged to practice PA by family members or by professionals themselves as a strategy for improving respiratory function.

However, the fear that vigorous physical activities may trigger asthma, combined with dyspnea due to lack of physical conditioning, and not exercise-induced bronchospasm itself, may cause some asthmatics and their parents to impose unnecessary limitations on physical activities.^1^ ^,^ ^2^ ^,^ ^18^ ^,^ ^19^ This lack of information is often accompanied by excessive caution from some teachers in guiding the practice of sports, which, when coupled with parents’ lack of confidence and fear of illness, may hinder their children’s inclusion in sports practice.^1^ ^,^ ^2^ ^,^ ^13^

In addition, individuals with asthma may be more susceptible to exercise-induced bronchospasm, as they already have pre-existing chronic inflammation in the airways, and exercise is the trigger for the hyperreactivity of the disease itself in individuals who are not under control.^20^ ^,^ ^21^ Thus, although they should be encouraged to practice PA, asthmatic patients may have greater restrictions on physical activities due to fear or lack of encouragement from health professionals, teachers, and family members.^1^ ^,^ ^2^ ^,^ ^13^

Research^5^ ^,^ ^22^ ^,^ ^23^ indicates that exercise may play a modulatory role in pulmonary inflammation by reducing bronchial hyperresponsiveness and the need for corticosteroids, with a reduction in the number of eosinophils in sputum and exhaled nitric oxide levels. The most recent Global Strategy for Asthma Management and Prevention (GINA),^11^ recommends prescribing PA as part of non-pharmacological treatment for asthma for general health benefits, so it should always be encouraged, including management and treatment of exercise-induced bronchospasm associated with appropriate medication. In addition, a detailed assessment of the daily activities of these young people should be included during the anamnesis to improve the diagnosis, such as the willingness to walk and play, and the importance of parental involvement to report irritability, fatigue, and children’s mood changes as an indicator of uncontrolled asthma.

A paper published^12^ by the American Thoracic Society and the European Respiratory Society about exercise programs in chronic diseases, including asthma, reports that training improves physical fitness and adds important effects in improving psychosocial aspects. Two randomized clinical trials^24^ ^,^ ^25^ have shown that having a physical training program improves symptoms, anxiety, depression, and quality of life in people with asthma, and an important systematic review^26^ complements that although exercise does not represent a significant improvement in lung function in children per se, it improves cardiorespiratory fitness and exercise-induced bronchospasm.

According to Battilani el al.,^27^ even with all the knowledge acquired on the practice of PA, asthmatic patients presented lower physical fitness compared to a non-asthmatic control group. In contrast, Dimitrakaki et al.^3^ and Eijkemans et al.^28^ reported that no difference in physical activity frequency was found among children with and without asthma. Wanrooij et al.^4^ concluded that the beneficial effects of physical exercise and the lack of negative effects indicate its recommendation in children with asthma. However, similarly to our research, one study^19^ reported that asthmatic children were more active than their peers and displayed favorable attitudes to PA practice. However, the authors hypothesized that this result was favored by an association with a current hype about the benefits of exercise for people with asthma. This fact was not investigated in our research, even so, no incentive campaign for PA aimed at adolescents with asthma was observed during data collection. Likewise, this higher PA practice frequency present in asthmatics does not exist in those with active asthma.

Question 6 of ISAAC, “Have you (your child) ever had asthma?”, evaluates asthma diagnosed by a physician and was used for this purpose according to standardized recommendations.^15^ ^,^ ^29^ From the responses to the ISAAC questionnaire, it can be inferred that asthma, at least on the part of physicians, was not neglected, as the frequency of its medical diagnosis was higher (17.9%) than that of wheezing in the last year (14%). However, this does not mean that patients are adequately cared for. This question assessing asthma diagnosed by a physician, along with health practices that value the practice of PA in adolescence, may have stimulated these patients to practice sports and PA.

In general, there is confusion with the question about chest wheezing so strong as to affect speech, which evaluates severe episodes of asthma and not the severe illness itself. In fact, lack of access or inappropriate use of anti-inflammatory drugs would have a potential effect on the severity of the disease. Because it was a research in which standardized and globally recognized questionnaires (ISAAC and IPAQ) were used,^15^ ^,^ ^16^ ^,^ ^17^ we did not have access to medication intake by adolescents, which may be one of the limitations of the study. Patients who had a history of more severe asthma attacks in the period between the attacks assessed by the study were as active as their peers (73.3 *versus* 63.5%; p=0.26).

This population-based study indicates the need for several possibilities for research on this topic, so that we can increasingly approach the real situation of asthma and PA in the patients affected.

However, a worrying finding was detected in this study: about 35% of the patients diagnosed with asthma and almost half of the adolescents studied without this diagnosis were inactive, identifying a fragility in the population monitoring of public incentive measures for the practice of physical activities. This pattern of likely sedentary lifestyle may lead to repercussions in the future.

One possible limitation of this study is that the questionnaires applied may be flawed as instruments for evaluation. However, these are validated and widely used in population surveys around the world, in addition to being able to subsidize future studies that can better characterize these asthmatic children. Another point to emphasize was the lack of research in health care units, in the schools and in the community itself about the existence of some policy or recommendation on the part of the professionals involved with these asthmatic adolescents on the incentive to PA practice.

Almost half of the children in the study were considered to be physically inactive. Adolescents diagnosed with asthma were more active than their non-asthmatic peers in this study, and this finding persisted when wheezing was present in the last 12 months and was independent of the history of a severe asthma attack in the study population. The influence of family members and health professionals in stimulating PA practice as part of asthma treatment is worthy of investigation.
